# Impact of the Disk Diffusion Test on Fluconazole De-Escalation in Patients with Candidemia

**DOI:** 10.3390/jof8111185

**Published:** 2022-11-10

**Authors:** Suchavadee Tantasuwan, Piriyaporn Chongtrakool, Amiroh Waesamaae, Methee Chayakulkeeree

**Affiliations:** 1 Department of Medicine, Faculty of Medicine Siriraj Hospital, Mahidol University, Bangkok 10700, Thailand; 2 Department of Microbiology, Faculty of Medicine Siriraj Hospital, Mahidol University, Bangkok 10700, Thailand; 3 Research Department, Faculty of Medicine Siriraj Hospital, Mahidol University, Bangkok 10700, Thailand; 4 Division of Infectious Diseases and Tropical Medicine, Department of Medicine, Faculty of Medicine Siriraj Hospital, Mahidol University, Bangkok 10700, Thailand

**Keywords:** candidemia, fluconazole, disk diffusion, antifungal susceptibility testing, *Candida*, antifungal de-escalation, antifungal stewardship

## Abstract

Disk diffusion (DD) is a simple antifungal susceptibility method for *Candida*. This study examined the impact of fluconazole DD testing on antifungal de-escalation. We enrolled patients with candidemia whose *Candida* isolates were tested for fluconazole susceptibility using DD between January 2019 and January 2020. The historical controls were patients with candidemia who underwent fluconazole susceptibility testing using the broth microdilution (BMD) method. Clinical data including antifungal therapy were analyzed. In total, 108 patients were enrolled. Most baseline characteristics were comparable between the groups. *C*. *tropicalis* was the predominant isolate (54.6%), followed by *C*. *albicans* (17.6%). The rates of antifungal de-escalation within 72 h were 25.9 and 9.3% in the DD and BMD groups, respectively (*p* = 0.023). The median time to de-escalation was 3 days in the DD group, versus 6 days in the BMD group (*p* = 0.037). The 14-day mortality rate and antifungal cost tended to be lower in the DD group. There were no differences in the length of hospital stay and treatment-related complications between the two groups. The agreement between the DD and BMD results was 90%. DD testing can be substituted for BMD to enhance antifungal de-escalation and antifungal stewardship.

## 1. Introduction

Candidemia is one of the leading causes of nosocomial bloodstream infection [1]. Although the diagnosis and treatment of candidemia have been improved, the mortality rate remains high [2]. Furthermore, this fungal bloodstream infection also increases the length of hospitalization and healthcare costs. *Candida albicans* is the predominant pathogen; however, the frequency of non-*albicans Candida* infection is increasing [3,4]. The prevalence of fluconazole-resistant *Candida* spp. remains relatively low, excluding *C*. *glabrata* and *C*. *krusei* [5]. Early available antifungal susceptibility testing is essential for selecting an appropriate antifungal therapy and enhancing antifungal stewardship programs [6,7].

According to the Clinical and Laboratory Standard Institute (CLSI) guidelines, the broth microdilution (BMD) test is a standard approved reference assay for determining the fungal susceptibility of *Candida* spp. [8]. Nonetheless, this method may not be practical in all clinical laboratories because it has a prolonged turnaround time and high costs. The disk diffusion (DD) antifungal susceptibility test is an alternative simple, rapid, inexpensive, and accurate method [9]. The CLSI has recommended the use of DD testing as a validated method to guide antifungal therapy [10].

In agreement with the current clinical practice guideline for managing candidiasis, echinocandin is recommended as the initial therapy [11]. In Thailand, because of the limited access to echinocandin, amphotericin B deoxycholate is commonly used as an alternative empirical antifungal treatment. Nephrotoxicity is the most common severe adverse effect related to amphotericin B therapy, and the associated mortality rate is high when acute kidney injury occurs [12]. A transition from empirical treatment to fluconazole is recommended for clinically suitable patients who have isolates that are susceptible to fluconazole.

There have been limited studies related to the use of fluconazole DD testing in clinical settings. The turnaround time of the DD antifungal susceptibility method is faster than that of the BMD method. We therefore hypothesize that the DD antifungal susceptibility method would facilitate effective early antifungal de-escalation and complement antifungal stewardship. In this study, we evaluated the fluconazole DD test as an alternative to the BMD method for assessing antifungal susceptibility and assisting clinicians in antifungal de-escalation in patients with candidemia.

## 2. Materials and Methods

### 2.1. Study Design

**Figure 1:**
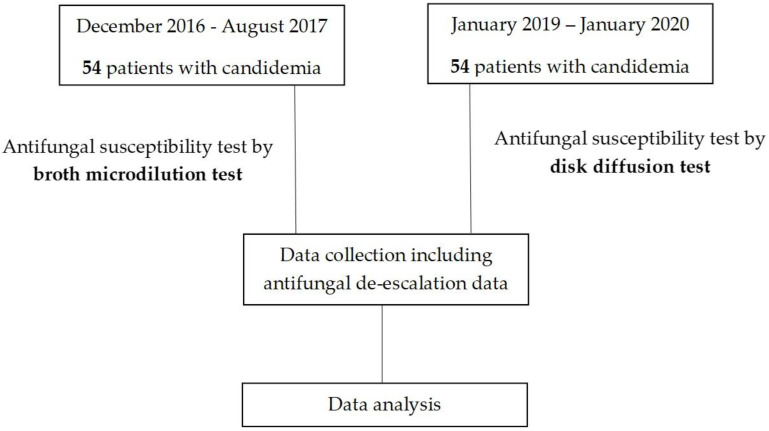
Study flowchart.

This was a prospective cohort study with a historical control. The mycology laboratory at our hospital routinely performs antifungal susceptibility tests using the BMD method. We enrolled adult patients with candidemia hospitalized at Siriraj Hospital, Mahidol University from January 2019 to January 2020. During this study period, the *Candida* isolates of the included patients were subjected to fluconazole susceptibility testing using the DD method. We also retrospectively reviewed the medical records of patients with candidemia treated between December 2016 and August 2017 who underwent antifungal susceptibility testing using BMD. Patients’ medical records were selected if they had positive blood cultures for *Candida* spp. The study flowchart is presented in Figure 1. This study was registered in the Thai Clinical Trials Registry (ID: TCTR20190501004).

### 2.2. Study Population

Patients aged ≥18 years with a positive blood culture for *Candida* were included. The exclusion criteria were as follows: receipt of fluconazole or no receipt of empirical antifungal treatment before antifungal susceptibility testing and terminal illness or expected death within 48 h after a diagnosis of candidemia. DD testing was performed according to the CLSI guidelines, and inhibition zone diameters were measured to determine susceptibility using the CLSI breakpoint [10]. Briefly, we used fluconazole disks containing 25 µg of fluconazole for DD testing. For *C*. *albicans*, *C*. *parapsilosis*, and *C*. *tropicalis*, an inhibition zone diameter of ≥17 mm indicated susceptibility to fluconazole, whereas an inhibition zone diameter of ≤13 mm indicated resistance. For *C*. *glabrata*, an inhibition zone of ≥15 mm was interpreted as fluconazole susceptible-dose dependent (SDD), and an inhibition zone of ≤14 mm was interpreted as drug resistant. BMD antifungal susceptibility testing was performed using the automated Sensititre^®^ method as a routine susceptibility test in our hospital.

The primary endpoints of this study were the rate of antifungal de-escalation within 72 h after a positive culture and the time to appropriate antifungal de-escalation. De-escalation of antifungal treatment was defined as a switch from initial antifungals (either amphotericin B or an echinocandin) to a triazole (such as fluconazole) within 72 h following their initiation. The secondary endpoints were 14-day mortality, 30-day mortality, length of hospitalization, total antifungal cost, and treatment-related complications.

The collected data included age, sex, clinical data at the time of candidemia diagnosis, comorbidities, length of stay after the diagnosis of candidemia, *Candida* species isolated from blood cultures, antifungal susceptibility test result, empirical antifungal treatment, clinical response, duration of treatment, total antifungal cost, time to culture negative, and antifungal de-escalation data.

### 2.3. Statistical Analysis

A statistical analysis was performed using PASW Statistic (SPSS) 18.0 (SPSS, Inc., Chicago, IL, USA). Subject characteristics were described using means, standard deviations, medians, frequencies, and percentages. The Student’s *t*-test and the Mann–Whitney *U* test were used for continuous variables, and the chi-squared test or Fisher’s exact test was used for discrete variables, as appropriate. *p* < 0.05 denoted statistical significance.

## 3. Results

### 3.1. Patient Data

A total of 108 patients were enrolled, 61.1% of whom were male, and the mean patient age was 61 ± 2.4 years. The baseline characteristics were similar between the groups, excluding the lower rate of prosthesis in the DD group (7.4% vs. 22.2%; *p* = 0.03, Table 1).

**Figure 2:**
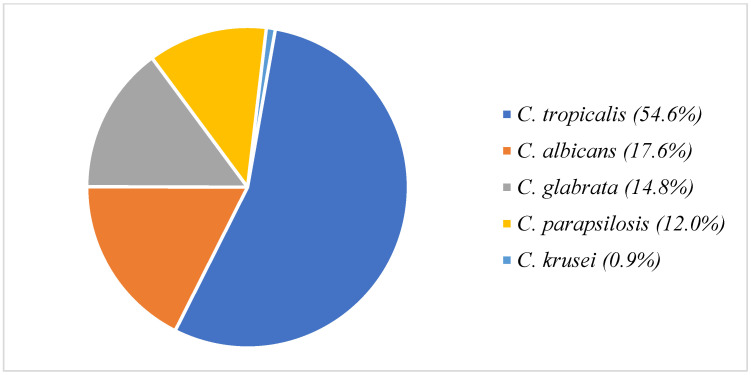
*Candida* isolates recovered in this study.

Of the *Candida* isolates, *C*. *tropicalis* was predominant (54.6%), followed by *C*. *albicans* (17.6%), *C*. *glabrata* (14.8%), *C*. *parapsilosis* (12.0%), and *C*. *krusei* (Figure 2). Fluconazole resistance was found in 13.3 and 16.7% of patients in the DD and BMD groups, respectively. Amphotericin B was used as the initial therapy in 48.1 and 66.7% of patients in the DD and BMD groups, respectively. The remaining patients received echinocandins as the initial therapy.

### 3.2. Antifungal De-Escalation

The rates of antifungal de-escalation within 72 h were 25.9 and 9.3% in the DD and BMD groups, respectively (*p* = 0.023). The median time to de-escalation was 3 days in the DD group, versus 6 days in the BMD group (*p* = 0.037). The 14-day mortality rates in these groups were 23.5 and 37.0%, respectively (*p* = 0.133). The median antifungal cost was 33,575 Baht in the DD group, compared to 48,470 in the BMD group (*p* = 0.621). There were no differences in the length of hospital stay and treatment-related complications between the two groups (Table 2). The rate of agreement of the fluconazole susceptibility results between the DD and BMD methods was 90%.

## 4. Discussion

Antifungal susceptibility testing is necessary for selecting an appropriate antifungal therapy, and it is a crucial part of antifungal stewardship. The standard BMD method has an extended turnaround time of approximately 72–96 h. Fluconazole DD susceptibility testing is a simple, rapid, inexpensive, and validated method. This procedure directly clarifies antifungal susceptibility using positive blood cultures. The turnaround time of this technique from the detection of blood culture positivity is 24–48 h. Furthermore, the fluconazole DD method is much cheaper than the BMD method. This was the first study in Thailand to investigate the roles of fluconazole DD susceptibility testing in the management of candidemia with a focus on antifungal de-escalation.

The study illustrated that non-*albicans Candida* was predominant, in line with its increased prevalence in Thailand in prior research [13]. Additionally, *C*. *tropicalis* was the most common *Candida* species, supporting previous reports [14,15,16]. Because of the increased prevalence of non-*albicans Candida*, fluconazole resistance was found in 13.3 and 16.7% of patients in the DD and BMD groups, respectively. In the historical control group, we found that amphotericin B was used as an empirical antifungal treatment in up to 66.7% of patients. However, because of recent changes in antifungal policy, echinocandin is more likely to be used as an initial therapy.

The results of our study indicated that fluconazole DD susceptibility testing led to early antifungal de-escalation in 25.9% of patients, versus 9.3% of patients using the conventional BMD method. Moreover, the DD method provided a shorter time to de-escalation (approximately 3 days) than the BMD test. These results suggest that DD susceptibility testing could significantly shorten the time to antifungal de-escalation. Regarding the secondary outcomes, namely 14-day and 30-day mortality, length of hospitalization, and treatment-related complications, there were no significant differences between the two groups. The median total antifungal cost tended to be lower in the DD group than in the conventional BMD group, although the difference was not statistically significant.

Antifungal de-escalation is a major tool for antifungal stewardship programs. Our study supports the previous studies in antifungal de-escalation and stewardship by Jaffal and Moreno-García [17,18]. Jaffal et al. have shown that antifungal de-escalation is safe and helps shorten the duration of antifungal therapy without affecting outcomes, including the duration of mechanical ventilation, length of ICU stay, and mortality [17]. In centers with increasing fluconazole-resistant *Candida* infection, empirical antifungal treatment with a broad-spectrum antifungal agent, either amphotericin B or an echinocandin, is suggested. However, once the antifungal susceptibility results become available, the de-escalation of antifungal therapy to fluconazole for fluconazole-susceptible *Candida* infection is strongly encouraged. A previous study revealed that an early stepdown from echinocandin to fluconazole treatment is safe in patients with candidemia caused by fluconazole-susceptible *Candida* and is important for antifungal stewardship strategies [18]. Furthermore, antifungal de-escalation has also been shown to have potential cost savings, associated with improved clinical success [19].

In the DD group, the inhibitory zone diameters were interpreted according to the CLSI document M60 [20]. We found that the rate of agreement of the fluconazole susceptibility results between the DD and BMD methods was 90%, consistent with a previous study in Thailand [21]. Furthermore, similar to our findings, recent studies have demonstrated that the DD antifungal susceptibility method showed a good agreement with the BMD method for *Candida* spp., including *C*. *glabrata* and *C*. *auris* [22,23]. However, the use of a larger inoculum may detect fluconazole-resistant *Candida* species more rapidly and reliably [24].

This study had some limitations that should be considered. This was a prospective cohort study with historical controls. There could be some differences in epidemiology and advances in medical care between the study periods, and the lack of blinding may have resulted in performance biases.

## 5. Conclusions

In conclusion, fluconazole susceptibility testing using the DD method provides a significantly higher rate of early antifungal de-escalation and a shorter time to de-escalation in patients with candidemia than BMD without affecting clinical outcomes. This simple and inexpensive DD test can be used in place of BMD to enhance antifungal de-escalation and antifungal stewardship.

## Figures and Tables

**Table 1:** Clinical characteristics of patients enrolled in the study.

Clinical Characteristics	DD Group (N = 54)	BMD Group (N = 54)	*p*-Value
Age (years), mean (SD)	61.18 (2.4)	61.89 (2.3)	0.236
Sex, Male	30 (55.6%)	36 (66.7%)	0.724
Clinical data at diagnosis of candidemia, N (%)			
Central venous catheter	38 (70.4%)	38 (70.4%)	1.000
Mechanical ventilator	33 (61.1%)	31 (57.4%)	0.695
Parenteral nutrition	25 (46.6%)	18 (33.3%)	0.169
Hemodialysis	17 (31.5%)	21 (38.9%)	0.420
Peritoneal dialysis	0	1 (1.9%)	1.000
Recent abdominal surgery	8 (14.8%)	9 (16.7%)	0.792
ICU admission	25 (46.3%)	27 (50.0%)	0.700
Urinary catheter	39 (72.2%)	40 (74.1%)	0.823
Presence of prosthesis	4 (7.4%)	12 (22.2%)	0.030
Receive carbapenem >7 days	26 (48.1%)	28 (51.9%)	0.700
Receive cephalosporin >7 days	9 (16.7%)	5 (9.3%)	0.252
Receive fluoroquinolone >7 days	5 (9.3%)	11 (20.4%)	0.104
Receive at least two ATB >10 days	13 (24.1%)	8 (14.8%)	0.224
Receive at least three ATB >10 days	3 (5.6%)	9 (16.7%)	0.066
Receive at least four ATB >10 days	8 (18.4%)	3 (5.6%)	0.112
Receive corticosteroids *	5 (9.3%)	10 (18.5%)	0.164
ANC < 500 cells/mm^3^	12 (22.2%)	10 (18.5%)	0.633
Presence of yeast in urine	25 (53.2%)	31 (67.4%)	0.162
Presence of yeast in sputum	21 (46.7%)	24 (52.2%)	0.599
Presence of yeast in feces	3 (14.3%)	5 (31.2%)	0.254
Prior antifungal exposure **	8 (14.8%)	12 (22.2%)	0.322
Prior azoles exposure **	7 (13.0%)	10 (18.5%)	0.428
Co-morbidities, N (%)			
Chronic cardiac disease	10 (18.5%)	11 (20.4%)	0.808
Chronic lung disease	3 (5.6%)	7 (13.0%)	0.184
Chronic kidney disease	14 (25.9%)	21 (38.9%)	0.150
Chronic liver disease	3 (5.6%)	6 (11.1%)	0.489
Diabetes mellitus	16 (29.6%)	17 (31.5%)	0.835
HIV infection	0	1 (1.9%)	1.000
Autoimmune disease	4 (7.4%)	4 (7.4%)	1.000
Hematological malignancies	11 (20.4%)	11 (20.4%)	1.000
Hematopoietic stem cell transplantation	2 (3.7%)	2 (3.7%)	1.000
Solid organ transplantation	1 (1.19%)	3 (5.6%)	0.618
Laboratory at diagnosis of candidemia			
AST (U/L)	30	37	0.353
ALT (U/L)	24	20	0.805
Creatinine (mg/dL)	1.69	1.25	0.783

Abbreviations: ICU, intensive care unit; ATB, antibiotics; ANC, absolute neutrophil count; HIV, human immunodeficiency virus; AST, aspartate transaminase; ALT, alanine transaminase; DD, disk diffusion; BMD, broth microdilution. * equivalent to prednisolone at a dose of least 20 mg/day for at least 2 weeks, ** in the past month.

**Table 2:** Primary and secondary outcomes of the study.

	DD Group (N = 54)	BMD Group (N = 54)	*p*-Value
Primary outcomes			
Proportion of antifungal de-escalation within 72 h, N (%)	14 (25.9%)	5 (9.3%)	0.023
Median time to antifungal de-escalation, days (IQR)	3 (2.0–5.0)	6 (3.0–9.0)	0.037
Secondary outcomes			
Fourteen-day mortality, N (%)	12 (23.5%)	20 (37.0%)	0.133
Thirty-day mortality, N (%)	20 (41.7%)	28 (53.8%)	0.223
Median length of stay, days (IQR)	17 (10.0–37.5)	15 (8.0–28.5)	0.239
Median antifungal cost, Baht (IQR)	33,575 (13,581–67,113)	48,470 (97,750–134,333)	0.621
Treatment-related complications, N (%)			
-Acute kidney injury	9 (29.0%)	10 (33.3%)	0.717
-Hypokalemia	23 (62.2%)	15 (45.5%)	0.161
-Hepatitis	6 (11.1%)	7 (13.0%)	0.767

Abbreviations: IQR, interquartile range; DD, disk diffusion; BMD, broth microdilution.

## Data Availability

The data presented in this study are available on request from the corresponding author. The data are not publicly available due to patients’ confidentiality.
